# Supplementary material for: Mutually exclusive sense–antisense transcription at FLC facilitates environmentally induced gene repression
Source: Nat Commun. 2016 Oct 7;7:13031. doi: 10.1038/ncomms13031 (PMC5059766; doi:10.1038/ncomms13031)
Supplement: Supplementary Information — Supplementary Figures 1-9 and Supplementary Tables 1-8 [file ncomms13031-s1.pdf]

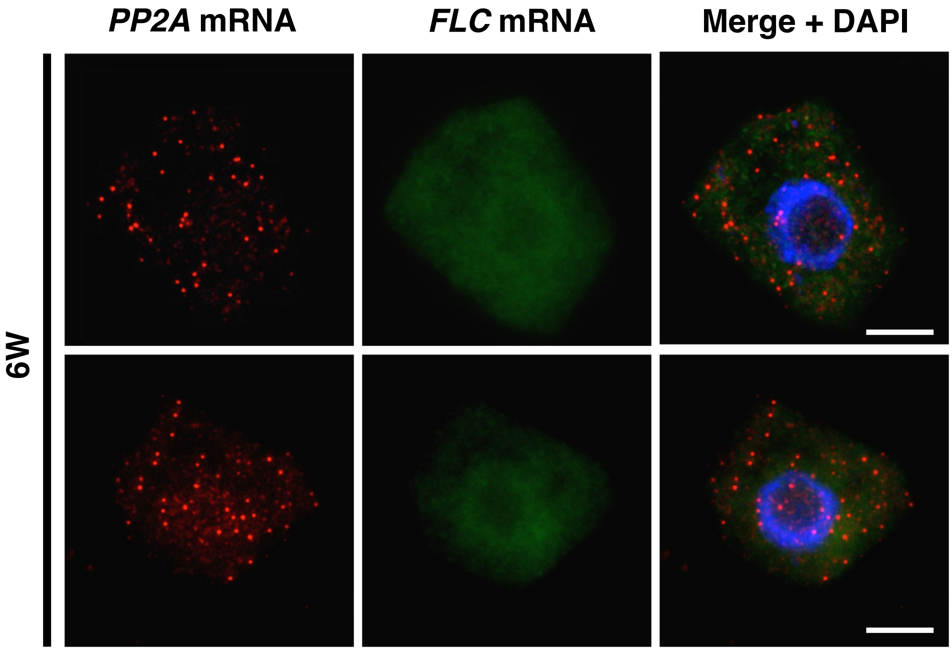

Supplementary Figure 1 – smFISH analysis for *PP2A* and *FLC* mRNA in 6 weeks vernalized sample. Representative images of cells hybridized with exonic smFISH probes against *FLC* (green) and PP2A (red) in plants vernalized for 6 weeks. *FLC* mRNA signals are absent, whilst *PP2A* is detected. DNA labeled with DAPI (blue). Scale bar: 5 µm.

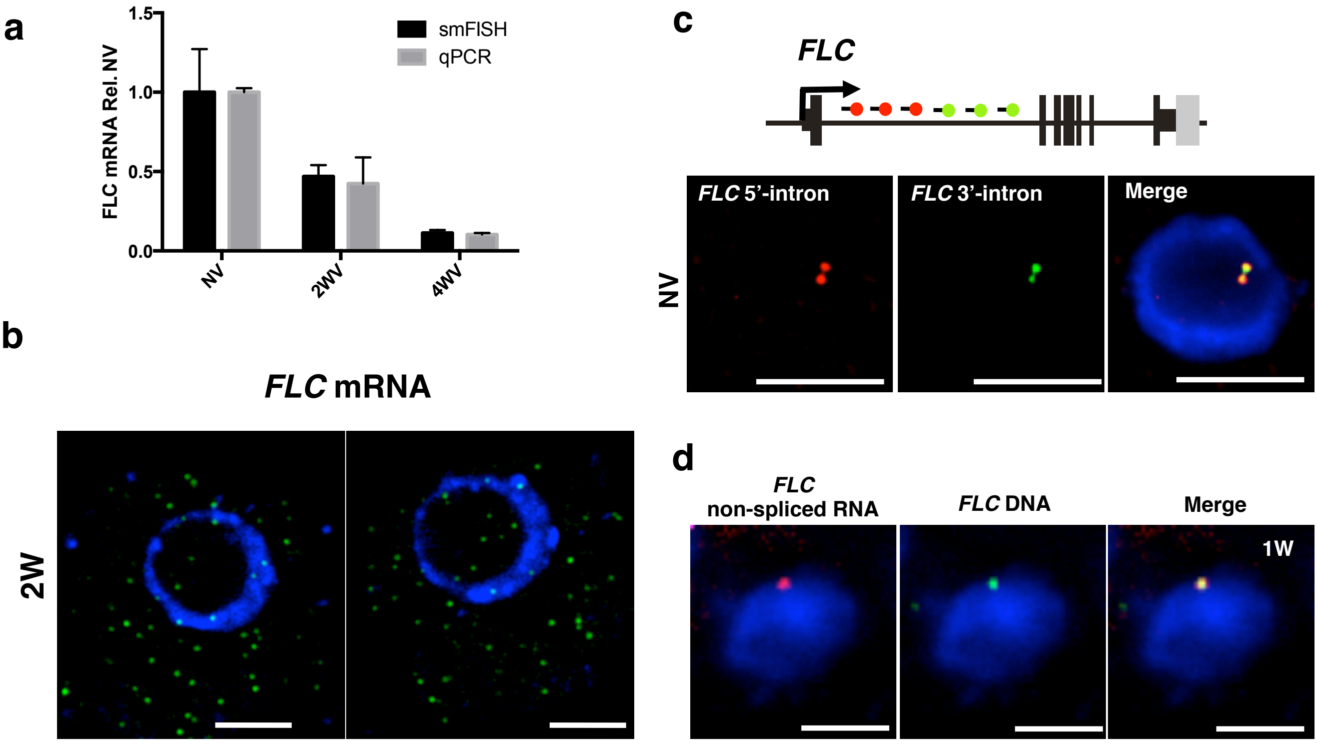

Supplementary Figure 2 – (a) Fold change in expression for *FLC* mRNA as measured by FISH and real-time RT-PCR (according to Duncan et al. 2015). Error bars are +/- SD. N=300 cells for smFISH and N=3 biological replicates for qPCR. (b) Representative images of cells hybridized with exonic smFISH probes against *FLC* (green) in plants vernalized for 2 weeks. No obvious localization of *FLC* mRNA is observed. (c) Schematic of the intronic probes used to detect the 5' (red) and 3' (green) end of *FLC* intron 1 and representative image showing co-localisation of 5'/3' signals. (d) Representative image of a nucleus showing colocalization of *FLC* intronic RNA smFISH signals (red) with *FLC* DNA FISH signals (green). DNA labeled with DAPI (blue). Scale bar: 5 µm.

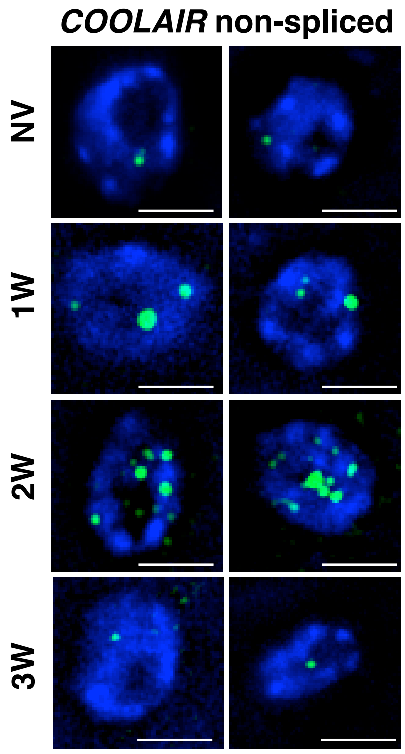

Supplementary Figure 3 – *COOLAIR* subcellular localisation. Additional examples of representative images of nuclei hybridized with intronic smFISH probes against *COOLAIR* (green) in non-vernalized plants (NV) and plants exposed to different weeks of cold (1W, 2W, 3W). DNA labeled with DAPI (blue). Scale bar: 5 µm.

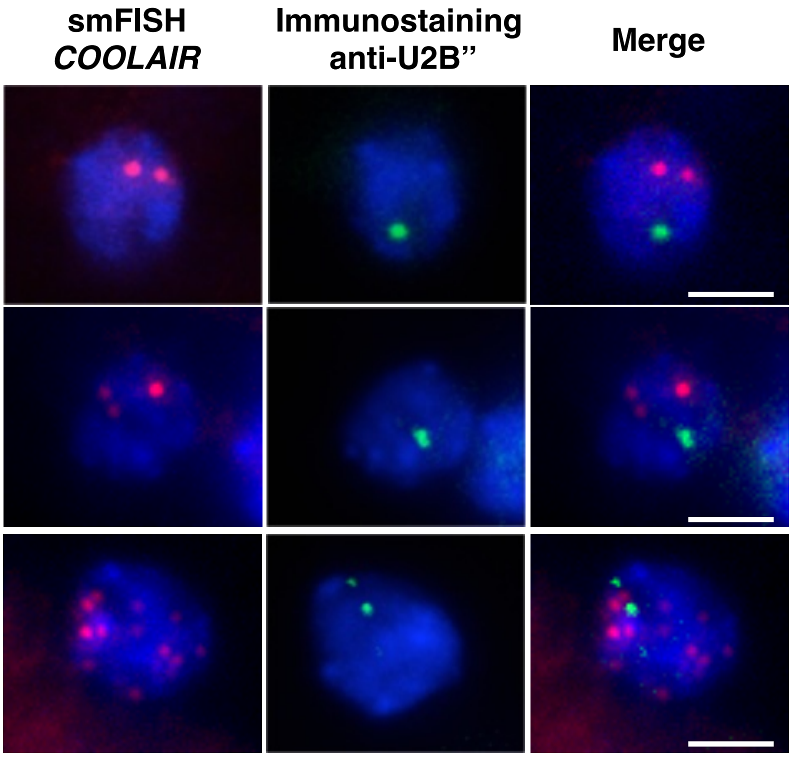

Supplementary Figure 4 – *COOLAIR* foci do not colocalize with Cajal Bodies. As we describe in the methods, we performed a protocol in which we combined Immunofluorescence and smFISH, here using an antibody against a Cajal body marker, the splicing protein U2B” (green), and intronic smFISH probes against *COOLAIR* (red). Nuclei from plants exposed to 2 weeks of cold. Scale bar: 5 µm.

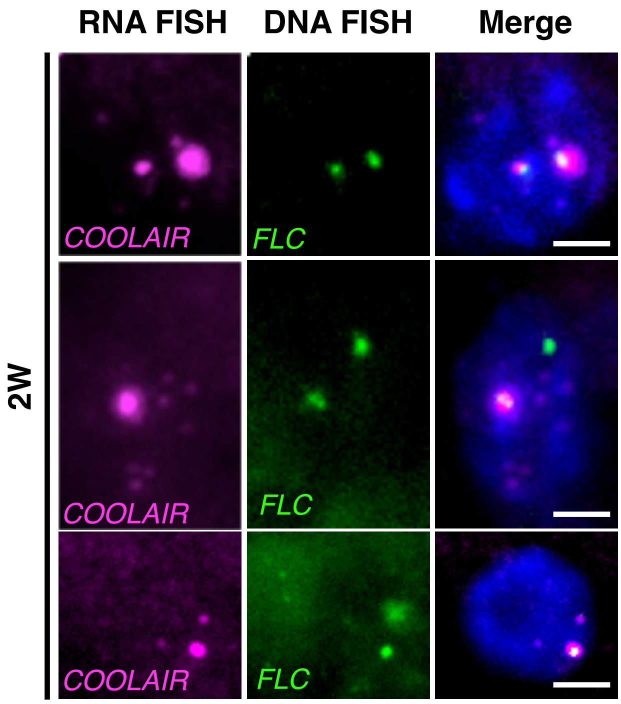

Supplementary Figure 5 – Colocalization of *COOLAIR* non-spliced RNA with *FLC* DNA. As we describe in the methods, we performed sequential RNA-DNA FISH, here we show additional examples of nuclei presenting colocalization of *COOLAIR* non-spliced RNA FISH signals (magenta) with *FLC* DNA FISH signals (green). Scale bar: 5 µm.

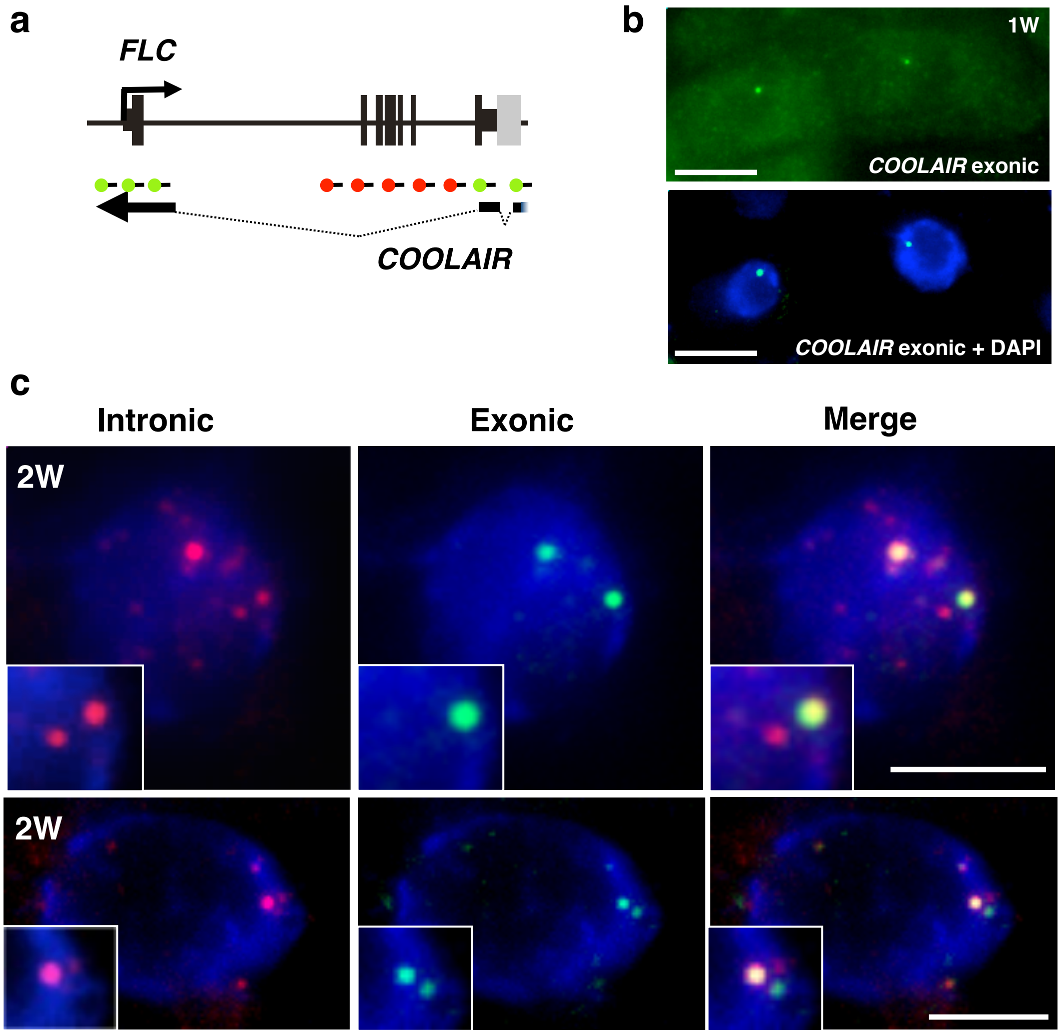

Supplementary Figure 6 – Double labeling with *COOLAIR* intronic and exonic probes. (a) Schematic of the probe locations used to detect *COOLAIR* introns (red) and exons (green). (b) Representative image of cells from plants exposed to 1 week of cold, hybridized with exonic smFISH probes for *COOLAIR* showing nuclear signal only. Scale bar: 10 µm. (c) Representative images of nuclei from plants exposed to 2 weeks of cold, hybridized with *COOLAIR* intronic (red) and exonic (green) probes. Strong colocalization was found with these two probe sets at large foci, but very little colocalization was observed at smaller foci. In most cases smaller foci showed intronic signals only (upper panel) but in some rare cases we observed small foci labeling only with exonic probes (lower panel). DNA labeled with DAPI (blue). Scale bar: 5 µm.

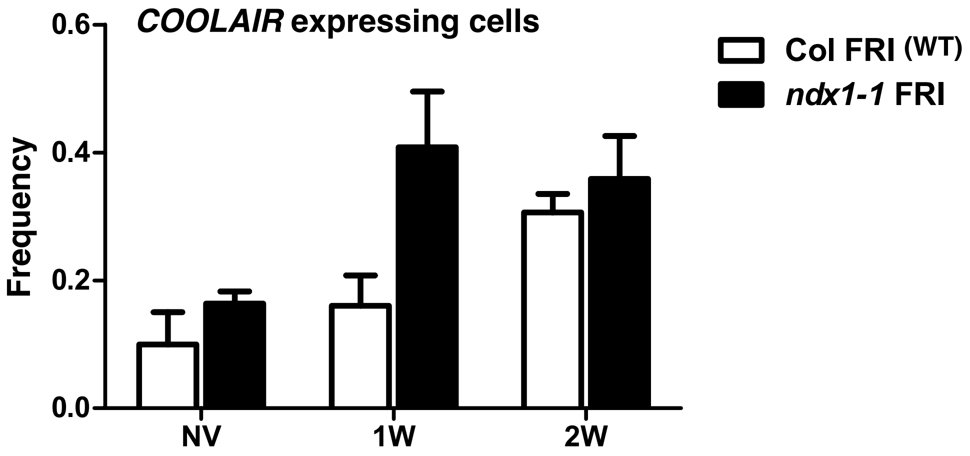

Supplementary Figure 7 – *ndx1-1* mutant shows increased number of *COOLAIR* expressing cells. Frequency of cells showing *COOLAIR* non-spliced signals with increasing cold exposure (NV, 1W, 2W) in wild-type ColFRI (WT) and *ndx1-1*FRI**.** Error bars are +/- SEM, with N=250 cells.

a
b
c

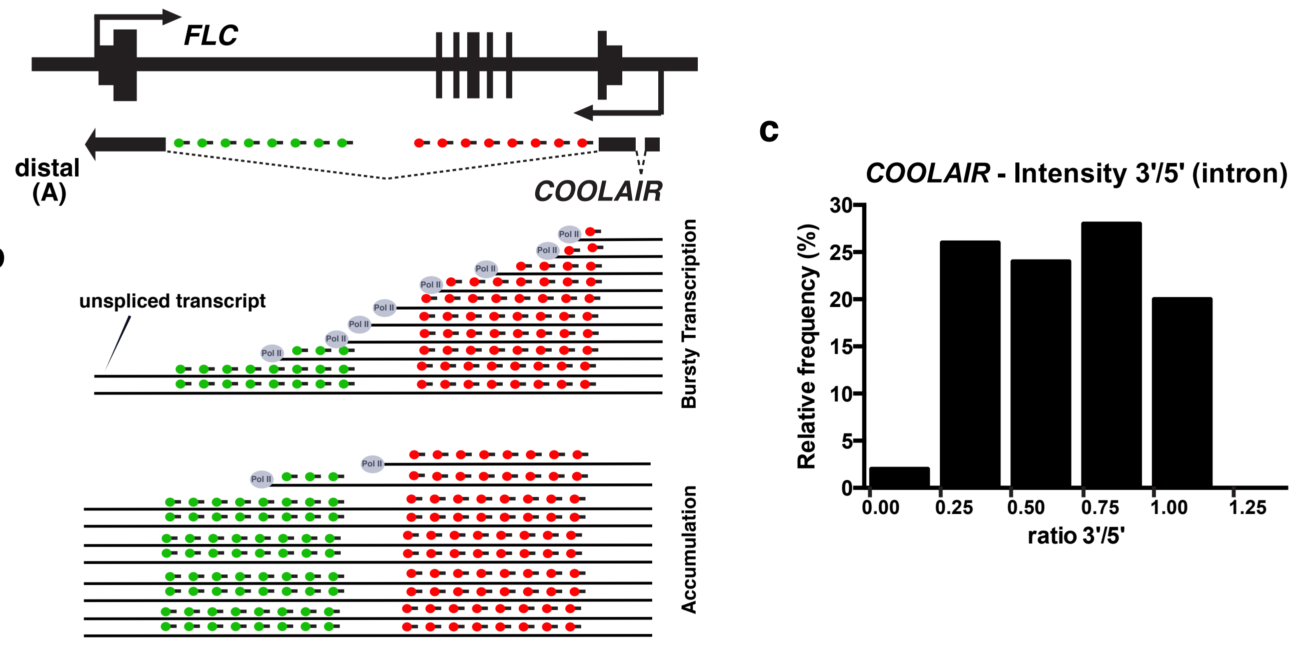

Supplementary Figure 8 – Schematic of double labelling experiment with 5' and 3' end probes for *COOLAIR* largest intron. (a) Schematic of the probes used to detect the 5' (red) and 3'-ends (green) of *COOLAIR* largest intron**.** (b) Two alternative scenarios for *COOLAIR* transcription (Bursty Transcription and Accumulation at the locus) and the expected labelling pattern. (c) Frequency distribution of 3'/5' total integrated intensity ratio for *COOLAIR* intronic probes (N=50 cells).

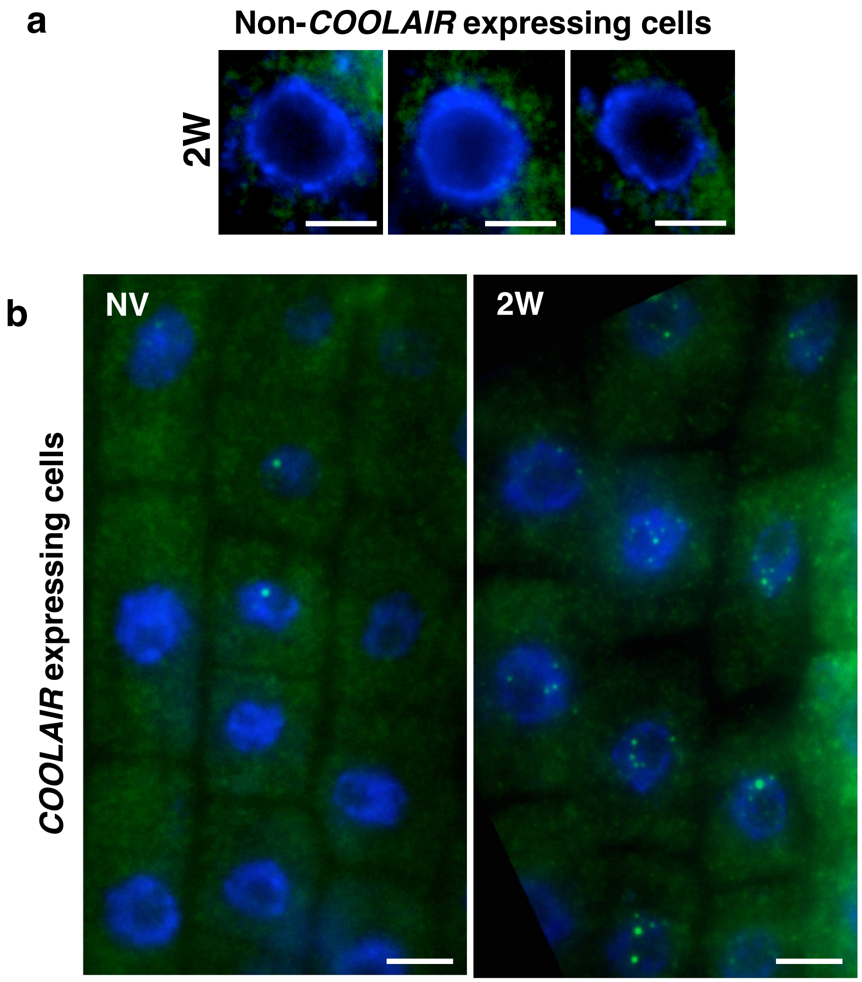

Supplementary Figure 9 – *COOLAIR* expression pattern. (a) Representative images of non-*COOLAIR* expressing cells hybridized with intronic smFISH probes against *COOLAIR* (green), in plants exposed to 2 weeks of cold. (b) Representative images of *COOLAIR* expressing cells (vasculature precursor cells) hybridized with intronic smFISH probes against *COOLAIR* (green), plants either non-vernalized plants (NV) or exposed to 2 weeks of cold (2W). DNA labeled with DAPI (blue). Scale bar: 5 µm.

Supplementary Table 1: smFISH probe sequences used to detect non-spliced *FLC* sense transcripts. These probes were labelled with Quasar570 and ordered from Biosearch Technologies.

**Sense *FLC* Intron 1 Probes**	**Sequences (5'-3')**
1	gatccgccggaaaaaaacca
2	catgtatctatcatggtcgc
3	cacgacattgttcttcctta
4	caacaacatcgagcacgcat
5	ctctatagatctcccgtaag
6	acattgttcagcattaaccc
7	caatagctgcacaatgtggt
8	aggtccacagcaaagatagg
9	aggctgagtttttgaagct
10	tgaagtagcatatgtgcggt
11	gcacacgacgattgtgattc
12	agaccagtttatgtacagca
13	tttataaatctcccggacgt
14	tcctttttaccattaacctc
15	tttcccaattaatgtggctt
16	gtgtaactgcaagagtggga
17	attgaggttgtggattgtca
18	ggttgtgtgattgtcgattt
19	atttcctagaggcaccaaag
20	tagatccgtaccaaagaggt
21	tggagggttgtagtagacac
22	gaccaacatggccaaactac
23	atcaagtgagaatcggccag
24	gacctaactaggggtgaaca
25	tagtcaggtgtctcgacaat
26	tccacgttctaaaaggcttc
27	gctctttgcatcaacctaag
28	gcccttgaagttacactaac
29	cggtcttccattttgttatt
30	tacatggaccgagtcttaga
31	gtatgaggaagccaaactcc
32	tgtggcggtaaccagataac
33	gctagtattgatgacccata
34	caaggtttttccagcgata
35	ggtaacatcagctctttgtt
36	aaacgcctctttcatgagtt
37	cttttcttttgtatcccag
38	acctatttaccctctatttt
39	ccataccacaacttttagca
40	agagattcagagcttccatt
41	tagtggaagactgcttccaa
42	ccaagtacacagactgagtc
43	gccacaatgtgatgacatgg
44	agccccaatcttaaatgcaa
45	tccagattgtttctatgcat
46	ccctaaacataagcctctac
47	tagcgctggctttgattaac
48	agcacatctgaatttccact

Supplementary Table 2: smFISH probe sequences used to detect *FLC* sense spliced. These probes were labelled with Quasar670 and ordered from Biosearch Technologies.

Sense *FLC* Exon Probes	Sequences (5'-3')
1	tttttttttccttttctcg
2	actaagcgttttctctttct
3	tcaggtttgggttcaagtcg
4	gctttgtgccctaatttgat
5	ctagtttttttcttcccatg
6	ttgttctcaattcgcttgat
7	gaaggtgacttgtcggctac
8	tgagaccgttgcgacgtttg
9	gaaagctgacgagctttctc
10	gacggatgcgtcacagagaa
11	gaggcggagacgacgagaag
12	gaggagaagctgtagagctt
13	aggatcttgaccaggttatc
14	atgctgtttcccatatcgat
15	ccaaggctttaagatcatca
16	agttcagagcttttgactga
17	agtagctcatagtgtgaacc
18	aagcttgctatccacaagtt
19	catttttgacatttgatccc
20	ccagttgaacaagagcatcg
21	agggcagtctcaaggtgttc
22	tcttcttggctctagtcacg
23	acaagcttcaacatgagttc
24	gcattttctccttttcttta
25	caaaacctggttctcttctt
26	atccaaggaatatctggcta
27	tcactttctctttttgtctt
28	ctccatctgtacgataatca
29	ctgctcccacatgatgatta
30	aggtgacatctccatctcag
31	agattgtcggagatttgtcc
32	taagtagtgggagagtcacc
33	tttcaaccgccgatttaagg
34	cccttatcagcggaataatt
35	ggccaaagagagagtattaa
36	agtatcacacacaaagtctc
37	agtattgacttagttccgtc
38	gttcatcaaccttttgtctt

Supplementary Table 3: smFISH probe sequences used to detect spliced *PP2A* transcripts. These probes were labelled with Quasar570 and ordered from Biosearch Technologies.

PP2A Exon Probes	Sequences (5'-3')
1	ccgagcgatctatcaatcag
2	gacatcctcaccaaaactca
3	tcgggtataaaggctcatca
4	tagctcgtcgataagcacag
5	ccaagagcacgagcaatgat
6	atcaactcttttcttgtcct
7	catcgtcattgttctcacta
8	atagccaaaagcacctcatc
9	atacagaataaaaccccccca
10	caagtttcctcaacagtgga
11	tcatctgagcaccaattcta
12	tagccagaggagtgaaatgc
13	cattcaccagctgaaagtcg
14	ggaaaatcccacatgctgat
15	atattgatcttagctccgtc
16	attggcatgtcatcttgaca
17	aaattagttgctgcagctct
18	gctgattcaattgtagcagc
19	ccgaatcttgatcatcttgc
20	caaccctcaacagccaataa
21	ctccaacaatttcccaagag
22	caaccatataacgcacacgc
23	agtagacgagcatatgcagg
24	gaacttctgcctcattatca
25	cacagggaagaatgtgctgg
26	tgacgtgctgagaagagtct
27	cccattataactgatgccaa
28	tggttcacttggtcaagttt
29	tctacaatggctggcagtaa
30	cgattatagccagacgtact
31	gactggccaacaagggaata
32	catcaaagaagcctacacct
33	ttgcatgcaaagagcaccaa
34	acggattgagtgaaccttgt
35	cttcagattgtttgcagcag
36	ggaccaaactcttcagcaag
37	ggaactatatgctgcattgc
38	gtgggttgttaatcatctct
39	tgcacgaagaatcgtcatcc
40	ttactggagcgagaagcga
41	ctctgtctttagatgcagtt
42	gaacatgtgatctcggatcc
43	catcattttggccacgttaa
44	cgtatcatgttctccacaac
45	atcaacatctgggtcttcac
46	ttggagagcttgatttgcga
47	acacaattcgttgctgtctt
48	cgcccaacgaacaaatcaca

Supplementary Table 4: smFISH probe sequences used to detect non-spliced *COOLAIR* transcripts (Intron 5'-end). These probes were labelled with Quasar570 and ordered from Biosearch Technologies.

Antisense *FLC* Intron Probes	Sequences (5'-3')
1	gtagtgctacttttacatgc
2	ttccaactccaagtgtctag
3	ttatggttaggtttggatcc
4	gtttatggaccgattagttt
5	agttaatcaccttttaacca
6	ggttgtgtaaacgttgtcta
7	gttcaatattggtttccttg
8	tggtctggttcagtctagtt
9	ttcttgattctctttcaggt
10	tctgggtttggtagagattc
11	gaaccttttatagtctggtt
12	ggttttggttcatttggaga
13	taggttttggttcttcttct
14	aattccggttgttggacata
15	acggctggttagagttaagg
16	gggttagtgagattattact
17	gttgttggtagtttggttta
18	ttcttctcaagattagggca
19	gtcttgtatagttgtattct
20	cattcactagttagcacttt
21	tatatagtcagtgcatttca
22	acactcttatgcttgcagat
23	atgtccatgtacatggacat
24	aataagcactgcgtgttgtg
25	acgaaagctacatttcctaa
26	acgaaagctacatttcctaa
27	attctgaagttgttaggttt
28	cttcatatgttttggattcc
29	gcttgcacacatatttgcaa
30	ctctgttactttaagtctgc
31	gatatatcctcttctgtgtt
32	gctaccaattttattgtaca
33	cgtgctgcttttgtttgttg
34	ctgaattttgtttgctgaga
35	atttcgtaatgtctactcct
36	ctctccacctttgattacaa
37	tcttctgtcccttttcatg
38	ttcatagcccttgtctttta
39	atgcattatgcataccgcaa
40	taaaatgaggtggtggctcc
41	actattagtttgccgagtga
42	atggagttttataaggcgta
43	aacattttgaatcttttccc
44	atttttttgtcatctctcc
45	agctagtagttttgatccta
46	tttccagtggccttttcaag
47	ggtgttctctcaatgtttca
48	gttacgaatactagcgtgtt

Supplementary Table 5: smFISH probe sequences used to detect exonic sequences in *COOLAIR* transcripts. These probes were labelled with Quasar670 and ordered from Biosearch Technologies.

**Antisense *FLC* Exon Probes**	**Sequences (5'-3')**
1	attttgcaacagggacgtgg
2	acagtgaagaagcctacggc
3	gtcaaaaacttgtgtttgct
4	gagtgtatgtgttcttcact
5	tcgtgtgagaattgcatcga
6	ggttgatgaactttgtacct
7	gagactttgtgtgtgatact
8	ttaatactctctctttggcc
9	taagggcgagcgtttgtata
10	agatatgtaattattccgct
11	accttaaatcggcggttgaa
12	gactctcccactacttaatt
13	aaatctccgacaatcttccg
14	tgagatggagatgtcacctg
15	atcatcatgtgggagcagaa
16	gattatcgtacagatggaga
17	gtgaatagtgattttgacct
18	tattccttggatagaagaca
19	gttgttatttggtggtgtga
20	accttctgtagtgtttttta
21	tttactttttactgcttcca
22	ccttttatcttctgttttgt
23	acctgggttttcatttgttc
24	gcgataagtacgccttttcc
25	aagctctacagcttctcctc
26	ttctgttctctgtgacgcat
27	tcatcgagaaagctcgtcag
28	ttctccaaacgtcgcaacgg
29	caaaagtagccgacaagtca
30	ggagagaagccatgggaaga
31	caaattagggcacaaagccc
32	acttgaacccaaacctgagg
33	aaaacgcttagtatctccgg
34	aaatatctggcccgacgaag
35	ctcgtttacccccaaaaaaa
36	tatttggtttttttgcatca
37	cgtggcaatcttgtcttcaa

Supplementary Table 6: smFISH probe sequences used to detect non-spliced *COOLAIR* transcripts (Intron 3'-end). These probes were labelled with Quasar670 and ordered from Biosearch Technologies.

Antisense *FLC* Intron Probes	Sequences (5'-3')
1	atgtcattttcaatctgccg
2	tgcctttgtatgacttttct
3	aagccagcgctatcactaaa
4	gtttagggttcttatgtacc
5	aatctggacagtagaggctt
6	ttgcatttaagattggggct
7	atcacattgtggctcatcaa
8	tttacttcggtttacttcca
9	tttctagactcagtctgtgt
10	aattggaagcagtcttccac
11	ttgttcgtagtcctgatcaa
12	gctggaaaaaaccttgtcct
13	tgggtcatcaatactagctc
14	gccacatcatcattatcatc
15	ttggcttcctcatacttatg
16	ctaagactcggtccatgtat
17	aaaatggaagaccggcttcc
18	attggaacctcacagtttct
19	ttatctgtcttagtcgcttc
20	ttgttggatcttctaggtca
21	tagaacgtggaacccttagt
22	gactagaactcctggtctta
23	ccttggaattgtcgagacac
24	catgttggtcaagatcgctg
25	tctggtgtagtgtctactac
26	ccacaacacttgtcttcatg
27	tttggtgcctctaggaaatt
28	tcttgtgtcttttgtcatgg
29	cttgtgtcttttgtcacaca
30	ccacaacctcaatcttttgt
31	tgactttgttcctattcgtt
32	tatcgcccttaatcttatca
33	acacacatagatttgcctca
34	aaggtttatagtttcccact
35	tcctttttatgggatatgct
36	gatttgtatatgcacgtccg
37	cacaatcgtcgtgtgctata
38	ggttatcgattgcgattctt
39	tacttaccgcacatatgcta
40	aattcctatctttgctgtgg
41	acattgtgcagctattgact
42	gctatggggttaatgctgaa
43	tgcacttacgggagatctat
44	attggatctctcggatttgt
45	tcttacctttagagattcct
46	gtgaagtttcaagccatctt
47	gcatgtcattcacgatttgt
48	gtttgtgtttttttctgcga

Supplementary Table 7: smFISH probe sequences used to detect *FLC* sense Intron1 5'-end. These probes were labelled with Quasar570 and ordered from Biosearch Technologies.

Antisense *FLC* Intron Probes	Sequences (5'-3')
1	ccaggtaaggaaaaggcgta
2	gatccgccggaaaaaaacca
3	catgtatctatcatggtcgc
4	gttcttccttaaatttggtt
5	gctaaaaagcttcttcacga
6	tccatgcagaattttttttt
7	tgacatgcaatttttttcca
8	aacatcgagcacgcatcaga
9	atggcttgaaacttcactca
10	tgcattgcatacaaatccga
11	aactctatagatctcccgta
12	acattgttcagcattaaccc
13	caatagctgcacaatgtggt
14	aggtccacagcaaagatagg
15	aggctgagtttttgaagct
16	tgaagtagcatatgtgcggt
17	tcgcaatcgataaccagatt
18	gcacacgacgattgtgattc
19	agaccagtttatgtacagca
20	tttataaatctcccggacgt
21	accattaacctcatactaat
22	accaaacttcttgatccttt
23	tttcccaattaatgtggctt
24	accaattcatcttttagtca
25	tctttaaatagcatatccca
26	ctgcaagagtgggaaactat
27	tgaggcaaatctatgtgtgt
28	attgaggttgtggattgtca
29	ggttgtgtgattgtcgattt
30	gatacaaaggttgtgtgaca
31	acaaggctgtgtgaatgaca
32	atttcctagaggcaccaaag
33	gacaagtgttgtgggatttt
34	gaggttggtatttcttacat
35	ttcattatagatccgtacca
36	caccagattcaattttgaca
37	tggagggttgtagtagacac
38	actacaaccatttggttatt
39	tcttgaccaacatggccaaa
40	atcaagtgagaatcggccag
41	gacctaactaggggtgaaca
42	gtgtctcgacaattccaagg
43	taagaccaggagttctagtc
44	gttctaaaaggcttcttctt
45	actgagtaactaagggttcc
46	gctctttgcatcaacctaag
47	atcagtcctattgtgaagtt

Supplementary Table 8: smFISH probe sequences used to detect *FLC* sense Intron1 3'-end. These probes were labelled with Quasar570 and ordered from Biosearch Technologies.

Antisense *FLC* Intron Probes	Sequences (5'-3')
1	ttgacctagaagatccaaca
2	tcttccatagaaggaagcga
3	gcccttgaagttacactaac
4	ttcctatatttaaaccccaa
5	atagaaactgtgaggttcca
6	cttccattttgttatttcac
7	ctcctaagaataggaagccg
8	tacatggaccgagtcttaga
9	gtatgaggaagccaaactcc
10	tgtggcggtaaccagataac
11	gctagtattgatgacccata
12	caaggtttttccagcgata
13	tcggatttttcaatgaacct
14	tcagctctttgtttgtaagt
15	atctcgtgtcaaaattggta
16	aaacgcctctttcatgagtt
17	cttttctttttgtatcccag
18	tcaggactacgaacaatggt
19	tttctatcatgtttaccctt
20	acaaaacctatttaccctct
21	gcgtgttatcaaaaccatta
22	aaacacctatatcctttaca
23	ccataccacaacttttagca
24	agagcttccattttggtttt
25	attgcaacctctatcagaga
26	tagtggaagactgcttccaa
27	agacttccctaacaatagca
28	ccaagtacacagactgagtc
29	gccacaatgtgatgacatgg
30	gcaatttttatttttccaga
31	agccccaatcttaaatgcaa
32	gcatttatgtttgtgaatct
33	tctactgtccagattgtttc
34	gttaaggtacataagaaccc
35	tagcgctggctttgattaac
36	gtcatacaaaggcatacaga
37	atgacattttccctcaaaga
38	atgtgggctaacatgtattt
39	gctcaaagattagttatcca
40	gatattttttttctctctca
41	tgttcttagctttgtaagca
42	tctgaatttccactttttca
43	ttcatgtttaagcagtagca
44	acacgctagtattcgtaaca
45	gagagaacaccttaccttttt
46	aggccactggaaactatgaa
47	aactactagctaacccttga
